# Multi-model assessment of yield stability in lentil across multi-environments

**DOI:** 10.1371/journal.pone.0356741

**Published:** 2026-09-01

**Authors:** Muhammad Jawad Asghar, Maria Ghaffar, Muhammad Shahid, Khalid Pervaiz Akhtar, Amjad Hameed, Aqsa Tabasum, Ghulam Rabbani, Uzma Javed, Muhammad Umar Shahbaz, Muhammad Kamran, Muhammad Ehtisham-ul-Haq

**Affiliations:** 1 Nuclear Institute for Agriculture and Biology (NIAB), Faisalabad, Punjab, Pakistan; 2 Barani Agriculture Research Institute (BARI), Chakwal, Punjab, Pakistan; 3 Plant Pathology Research Institute, Ayub Agricultural Research Institute (AARI), Faisalabad, Punjab, Pakistan; 4 Sugarcane Research Institute, Ayub Agricultural Research Institute (AARI), Faisalabad, Punjab, Pakistan; University of Agriculture Faisalabad, PAKISTAN

## Abstract

Developing high-yielding and stable lentil genotypes is essential for sustaining productivity under increasingly variable climatic conditions. However, strong genotype × environment (G × E) interactions often complicate the identification of widely adapted genotypes. Although several stability models are available, each has distinct strengths and limitations, making it uncertain whether a single model or a combination of models provides the most reliable selection. Therefore, this study compared multiple stability models to identify high-yielding and stable lentil genotypes and to evaluate their effectiveness for genotype selection. A set of lentil genotypes was evaluated across four diverse environments using a randomized complete block design (RCBD). Adjusted mean data were analyzed using the Eberhart and Russell regression model, AMMI (Additive Main Effects and Multiplicative Interaction), BLUP (Best Linear Unbiased Prediction), and WAASBY (Weighted Average of Absolute Scores and Yield). Significant G × E interactions confirmed differential genotype responses across environments. The Eberhart and Russell model identified stable genotypes based on regression coefficients close to unity and minimal deviation from regression. AMMI and BLUP effectively partitioned G × E effects and distinguished genotypes with broad and specific adaptation. WAASBY integrated yield and stability into a single index and was particularly effective in identifying genotypes with superior performance and wide adaptability. Correlation analysis among stability indices and the Genotype Selection Index (GSI) further strengthened the comparison and integration of model outputs. Genotypes G4, G8, G9, and G3 consistently exhibited high yield and stable performance across environments, with G4 emerging as the most promising and widely adapted genotype. This study demonstrates that integrating regression, mixed model, and multivariate based stability analyses provides a more robust framework for selecting stable, high-yielding lentil genotypes than relying on a single method, thereby supporting breeding for diverse agro-ecological conditions in Pakistan.

## Introduction

Lentil (*Lens culinaris* Medikus) is an important cool-season, rain-fed food legume and a key crop for diversifying cereal-based farming systems worldwide. In Pakistan, it is among the four major pulse crops, valued for its high protein content (around 25%) and its significant role in the cereal-based diet of the population [1]. However, despite rising demand driven by population growth, its cultivated area and production in the country have declined sharply compared to previous decades. This low productivity is closely related to the effects of climate change, which raises the risk of extreme weather, temperature increases, and ecological disruptions that have a negative influence on capital productivity, agricultural output, and overall economic performance. As climate change is predicted to increase abiotic stress and cause more severe disease epidemics, as seen with chickpea *Ascochyta* blight, along with increased weed pressure, pests’ attacks and change in life cycle of pathogens, low production will become a significant issue [2]. These challenges emphasize the urgent need to develop climate-resilient cultivars that can sustain yields and support food security. This requires not only selecting high-yielding genotypes but also identifying those well adapted to specific environments and mega-environments [3,4]. Unstable environmental conditions caused by climate change make stability analysis essential for developing adaptation strategies that safeguard long-term agricultural and economic sustainability.

Stability analysis plays a critical role in plant breeding by assessing the consistency of genotype performance under variable and often unpredictable conditions. Its primary goal is to identify genotypes that exhibit a stable response across environments while maintaining high mean yield [5,6]. Such dual emphasis on yield and stability enables the development of resilient cultivars capable of performing reliably across different regions and climatic scenarios. Accurate prediction of yield is essential for the success of new varieties. Multi-location trials are therefore a cornerstone for identifying genotypes that are either specifically adapted to particular environments or broadly stable across a range of conditions [7,8]. The effectiveness of such studies depends both on the accuracy of experimental estimates and the capacity to predict performance in new environments [9,10].

Crop breeding benefits from the application of numerous stability models since no single model can adequately explain genotype performance across multiple environments [11]. Using different approaches allows cross-validation, enhances reliability, and provides a broader understanding of genotype x environment interactions. In order to ensure well-supported selection judgements, this integrated approach is especially beneficial for complicated datasets and a variety of breeding materials [12,13]. New and sophisticated models are being developed to better comprehend the genotype x environment link since genotype stability analysis for yield has now become a crucial component of plant breeding programs. Different statistical techniques are used by each model to describe genotype-by-environment interactions, providing distinct insights, i.e., regression based, Anova based, BLUP based etc. Models such as Eberhart and Russell or the coefficient of variation evaluate yield consistency and responsiveness, whereas AMMI and GGE bi-plot efficiently understand interaction patterns and distinguish environments.

Among the earliest methods, Stringfield and Salter [14] described environmental responses using a linear regression coefficient (b), which was later expanded by Eberhart and Russell (1966) into a comprehensive regression model. They proposed a comprehensive linear regression model that suggested the use of regression coefficient (b) and mean square deviation (S^2^di) as a stability parameter for describing response of genotypes across environments [15]. Although widely applied, this approach assumes linear responses to environmental indices, which may not adequately capture complex genotype-environment interactions.

The Additive Main Effects and Multiplicative Interaction (AMMI) model is another commonly used approach, efficiently partitions main and interaction effects, making it a powerful tool for quantifying GEI and identifying stable, adaptable genotypes [16,17]. This is a hybrid model which increases estimation accuracy in two steps, where standard ANOVA is used to separate additive and multiplicative variance (GEI) and then the Principal Component Analysis (PCA) is performed to withdraw pattern of interactions from GE part of ANOVA. However, the AMMI1 bi-plot has limitations in assessing the discriminating ability and representativeness of environments.

To address this, Yan et al. [18], building on Gabriel [19] proposal, introduced the GGE bi-plot, which graphically displays and interprets genotype main effects and GEI in METs, and has since proven effective for exploring these two key sources of variation; genotype main effect and GEI [20,21]. This graphical representation of numerical data allows drawing a straightforward interpretation of fundamental cause of the GEI and summarizes the pattern present in raw data. Yan and Kang [22], claim that it also facilitates the selection of better genotypes and the identification of mega-environments, which are collections of test environments that share the same best-performing genotype (s). However, GGE bi-plot may capture only a small portion of total G + GE variation when genotype effects are minor relative to G × E interaction or when interaction patterns are highly complex, thereby limiting its accuracy in certain multi-environment trials [23].

More recently, Best Linear Unbiased Prediction (BLUP) has been widely adopted in MET analysis. BLUP is a robust statistical method that accounts for random effects and environmental variation, providing precise estimates of genotype performance and genetic values, even in complex multi-environment trials. Even in intricate multi-environment trials and mixed linear models, it provides great accuracy in genotypic performance prediction. To integrate the strengths of AMMI and BLUP, Olivoto et al. [24] introduced the weighted average of absolute scores of BLUPs (WAASB), which summarizes multiple interactions across principal component axes (IPCAs) and provides reliable estimates of stability for ranking genotypes. Recognizing that breeders must consider both yield (Y) and stability (WAASB) simultaneously when releasing new varieties, the WAASBY index was further proposed. This index combines mean yield (Y) with stability (WAASB), allowing breeders to adjust the weight given to each trait according to breeding objectives and varietal recommendations, thus facilitating the selection of cultivars with both high productivity and broad adaptability.

Despite substantial progress in lentil stability analysis, most studies still evaluate genotype performance using a limited number of stability models, which may not fully capture the complexity of genotype x environment interaction or identify genotypes that are both high yielding and widely adapted. Recent lentil investigations have shown that different methods can rank genotypes differently and that integrated approaches are more effective for selecting stable genotypes under variable conditions [5,7,25]. Therefore, a comparative multi-model assessment is needed to improve selection accuracy and provide stronger breeding recommendations for diverse environments [4,26]. Given that each model has unique strengths and limitations, the present study was designed to i) evaluate the performance and stability of lentil genotypes across environments, ii) to compare the predictive value of different stability models, and iii) to identify the most reliable approach for future breeding studies.

## Materials and methods

### Experimental design and field evaluation

**Fig 1:**
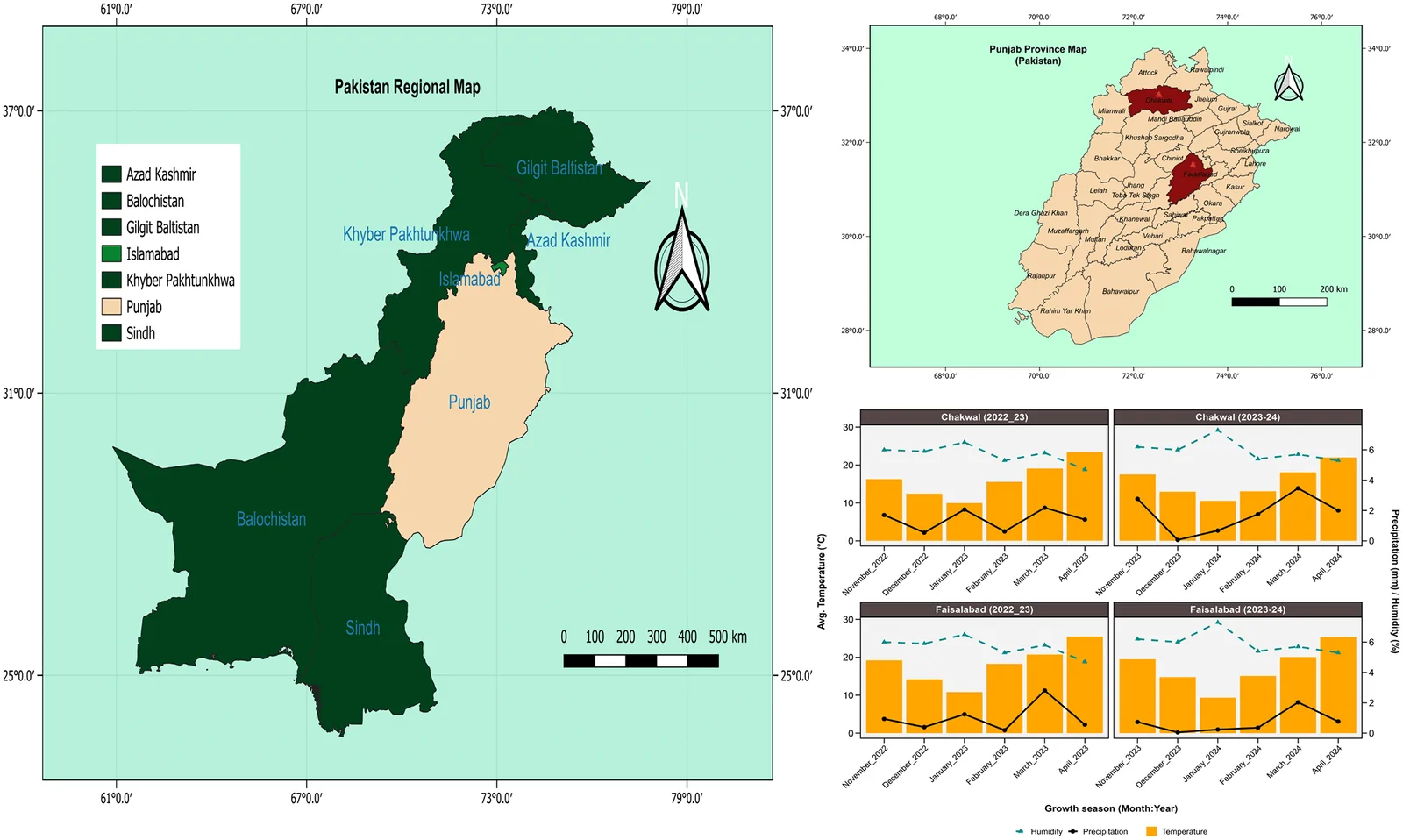
Geographic distribution of the experimental locations in Punjab, Pakistan, together with monthly weather conditions during the 2022–23 and 2023–24 growing seasons. The map was created by the authors in QGIS Desktop (version 3.36.1) using administrative boundary data from the GADM database (version 4.1). No proprietary map or satellite imagery was used.

This study assessed ten elite lines of lentil (*Lens culinaris* Medik.) (**Table 1**) that were developed from local lentil germplasm at the Nuclear Institute for Agriculture and Biology (NIAB), Faisalabad, Pakistan through hybridization and mutation breeding (chemical and physical). Four cultivars served as controls. The METs were conducted over the two growing seasons of 2022-2023 and 2023-2024 at two sites in Punjab province, Pakistan (Fig. 1). During land preparation prior to sowing, the recommended doses of NPK (50:25:20 kg ha^-1^) in form of DAP (Diammonium Phosphate) and SOP (Potassium Sulphate) were uniformly applied as basal. A randomized-complete block design (RCBD) with three replications was used to set up the experiment. Every plot had four rows with a typical 30 cm gap between rows and a 10 cm gap between plants. After two weeks of emergence, the plants were thinned to one plant per hole to maintain the population size across the experiment. Seed yield per plot (4 m × 1.2 m = 4.8 m^2^) data were collected from both locations across growth seasons and converted into kg ha^-1^.

**Table 1:** List of advance lines contributed to study at two locations across two growing seasons at Punjab Province, Pakistan.

Code	Genotypes	Nature	Pedigree/ Parentage
G1	LPP 21113	Mutant	M_6_ (LPP 12137/125 Gy)
G2	LPP 21114	Mutant	M_6_ (LPP 12137/125 Gy)
G3	LPP 21104	Hybrid	F_6_ (LPP 12137 × LPP 12198)
G4	LPP 21116	Mutant	M_5_ (PM 2020/0.1% SA)
G5	LPP 21112	Mutant	M_6_ (LPP 12137/125 Gy)
G6	LPP 21110	Mutant	M_6_ (LPP 12137/100 Gy)
G7	LPP 21120	Hybrid	F_6_ (12017 × 12191)
G8	LPP 21125	Mutant	M_5_ (LPP 12161/0.1% SA)
G9	LPP 21123	Hybrid	F_4_ (LPP 11025 × PM 2020)
G10	LPP 21132	Mutant	M_6_ (LPP 12191/0.15% SA)
S1	PM 2020	Standard check-1	–
S2	NL-2002	Standard check-2	–
S3	Markaz 2009	Standard check-3	–
S4	Chakwal Masoor	Standard check-4	–

### Statistical analysis

Environments were defined as location × year, hence a total of four environments were identified, i.e., FSD1 & CHK1 for year-1 and FSD2 & CHK2 for year-2. Analysis of variance (ANOVA) of each environment and pooled ANOVA was done according to Gomez and Gomez [27]. Genotypes response across individual environments was assessed employing one-way analysis of variance (ANOVA) and the results were found significant across individual environments. Based on this, two-factor pooled ANOVA was done to identify the interaction between genotypes and environments at p < 0.05. Bartlett’s test was applied to the pooled yield data across all environments to check the homogeneity of error variance. Following the initial analysis, the stability models and indices (Eberhart and Russell model regression model, AMMI, GGE and WAASBY) were applied. After model application, GSI was calculated to get a single cumulative value and ranking. Moreover, Spearman’s rank correlation was calculated to identify the degree of association between models’ ranks. Genetic parameters: GCV, PCV, h2, GA were also calculated for genotypes to aid the selection. Based on the GSI and genetic parameters the stable high yielders were selected. All the model application, analysis and the graphs/plots were generated with R software (version 4.4.3) using packages: *metan, ggplot2, variability, EstimateBreed*. The models applied were as follows:

### Eberhart and Russell’s model

Eberhart and Russell (1966) model of stability was used to study the non-linear (S²d) and linear (b) parameters of phenotypic stability. The Eberhart and Russell model is given as:


$$\begin{array}{c} Yij=m+biIj+\delta \text{ ij}+\epsilon \text{ij} \end{array}$$

Where Yij = Mean of i^th^ genotype in j^th^ environment, m = Mean of all genotypes in all environments, bi = Regression coefficient of i^th^ genotype, Ij = Environmental Index, which is defined as the mean deviation from overall mean for all genotypes at a given location, δ ij = Deviation from Regression of i^th^ genotype at j^th^ environment and εij is the mean experimental error. Genotypes were considered fixed, while environments were random variables. This model provides two stability parameters; i) linear regression coefficient (bi) of genotype means on all genotypes mean in each environment, ii) mean squares of deviation from regression (S^2^di) for each genotype, calculated as:


$$\begin{array}{c} bi=\text{1}+\frac{\sum (Xij-Xi-Xj+X..)(Xj-X)}{\sum (Xj-X)} \end{array}$$

$$\begin{array}{c} S^{\text{2}}di=\frac{\text{1}}{E-\text{2}}\left[\sum i(Xij-Xi-Xj+X..)(Xj-X)-(bi-\text{2}{)}^{\text{2}\sum j}(Xj-X..{)}^{\text{2}}\right] \end{array}$$

Where X_ij_ = seed yield of i^th^ genotypes and j^th^ environment, X_i_ = mean seed yield of i^th^ genotype, X_j_ = mean seed yield of j^th^ environment, X = overall mean and E is the total number of environments studied. Rating of each genotype was done based on mean yield, deviation from regression line (S^2^di=0) and regression co-efficient (b = 1). Genotype with non-significant deviation from regression (S^2^di=0) and significant unit regression-coefficient (b = 1) was considered as stable. The hypothesis testing was done using pooled error to see if the mean square deviation did not differ significantly from 0 at 0.05 probability level. F-test was applied to measure the significance of S^2^di and t-test was applied to measure the significance of bi. The t-test tested the hypothesis using the standard error of regression coefficient (SE_b_) that bi did not differ from unity. The ranking was done based on stability rating and mean yield separately, and then a cumulative rank was calculated for final ranking of genotypes.

### AMMI analysis

AMMI bi-plot analysis [28] was conducted to evaluate seed yield variation due to genotype and environment and to assess lentil variety adaptation using the first two principal components. The AMMI1 bi-plot plotted means (genotype × environment) against IPCA1 scores on x-axis and y-axis respectively, while AMMI2 used IPCA1 (x-axis) and IPCA2 (y-axis) scores. These bi-plots effectively illustrate GEI, with PC1 and PC2 typically explaining over 50% of the interaction variance (Crossa & Cornelius, 1997). The AMMI model is expressed as:

t


$$\begin{array}{c} Yij=\mu +\alpha i+\beta j+\Sigma \lambda \kappa \xi ik\overset{´}{\eta }jk+\varepsilon ij \end{array}$$

k = 1

where εij is the model’s error term, t is the number of single value decomposition (SVD) axes used in the model, 𝜆𝜅 = singular value for the SVD axis k, 𝜉𝑖𝑘 = singular value of the i^th^ genotype for the SVD axis k, and ήjk = singular value of the j^th^ environment for the SVD axis k. AMMI Stability Index (ASI) was computed as a stability parameter based on AMMI results [29]. ASI combines the contribution of interaction IPCAs from the AMMI model to provide a numerical stability measure for each genotype. Genotypes were ranked based on their ASI values; lower ASI values indicate greater stability across environments. ASI was calculated using the following formula:


$$\begin{array}{c} ASIi=\sqrt{(IPCA\text{1}i{)}^{\text{2}}+(IPCA\text{2}i{)}^{\text{2}}} \end{array}$$

Where ASIi = AMMI Stability Index for the i^th^ genotype, IPCA1i and IPCA2i = Score of the i^th^ genotype in the 1^st^ and 2^nd^ Interaction Principal Component Axis. Moreover, Weighted Average of Absolute Scores (WAAS) [24] were also calculated and genotypes were ranked from lower to higher values, with lower being more stable one. The WAAS was computed by the following formula:


$$\begin{array}{c} WAAS_{i}=\;\frac{\sum_{k=\text{1}}^{p}|IPCAik\;\times EPk|}{\sum_{k=\text{1}}^{p}EPk} \end{array}$$

where IPCAik represents the genotype’s score in the k^th^ Interaction Principal Component Axis, and EPk is the explained variance of that axis.

### GGE-Biplots

The GGE (Genotype and Genotype × Environment) bi-plot [18] was also employed to identify high-yielding, stable, and widely adaptable genotypes across environments. The GGE model was applied using following formula [30]:


$$\begin{array}{c} Yij\;=\;\mu \;+\;Ej\;+\;Gi\;+\;GEij\;+\;eij \end{array}$$

where Ej stands for random error, Gi for genotypic effect, GEij for genotype × environment effect, μ for population mean, and eij for environmental effect. Ranking genotypes and which-won-where bi-plots were developed after Single Value Partitioning (SVP) of the genotypes and environmental data. Genotypes ranking was done based on their contribution to dimension 1 and 2.

### WAASBY (based on BLUPs)

Best Linear Unbiased Predictor (BLUP) values for yield data were calculated. The Weighted Average of Absolute Scores for BLUPs (WAASB) and Weighted Average of Absolute Scores and Yield (WAASBY) are statistical indices proposed by Olivoto et al. [24] to evaluate genotype stability and performance in multi-environment trials (METs). WAASB quantifies stability using the formula:


$$\begin{array}{c} WAA{SB}_{i}=\;\frac{\sum_{k=\text{1}}^{p}|IPCAik\;\times EPk|}{\sum_{k=\text{1}}^{p}EPk} \end{array}$$

where IPCAik represents the genotype’s score in the k^th^ Interaction Principal Component Axis, and EPk is the explained variance of that axis. To select genotypes the WAASBY index, which is a superiority index was calculated, that combine high performance (mean yield) and stability (WAASB) and allows weighting between both parameters. The first step involves rescaling both seed yield (SYLD) and WAASB values to a 0–100 scale for direct comparison. Since a higher SYLD is desirable, while a lower WAASB indicates greater stability, the rescaling was performed using the following formulas:


$$\begin{array}{c} Yi=\frac{\text{1}\text{0}\text{0}-\text{0}}{Ymax-Ymin}\times (Yi-Ymax)+\text{1}\text{0}\text{0} \end{array}$$

$$\begin{array}{c} Wi=\frac{\text{1}\text{0}\text{0}-\text{0}}{Wmax-Wmin}\times (Wi-Wmax)+\text{1}\text{0}\text{0} \end{array}$$

where *rYi* and *rWi* are the rescaled values for SYLD and WAASB, respectively, for the *i*t^h^ genotype; *Yi* and *Wi* are the response variable (SYLD) and the WAASB values for *i*^th^ genotype. WAASBY integrates both stability and mean yield performance through the equation:


$$\begin{array}{c} WAASBYi=\frac{\left(rYi\times \theta Y)+(rWi\times \theta s\right)}{\theta Y+\theta s} \end{array}$$

where rYi and rWi are rescaled values for yield and stability, and θY, θs are user-defined weights. Weights for yield and stability can be allotted as per breeders’ priority based on the trait under consideration and varietal recommendations. The genotype with the highest WAASBY score would be attributed with a first-order rank. These indices enable plant breeders to balance yield and stability during selection, with WAASBY offering flexibility to prioritize traits (e.g., 70% yield, 30% stability). By combining singular value decomposition (SVD) of genotype-environment interactions with weighted rankings, WAASBY improves the identification of robust genotypes for diverse agricultural conditions. In our study, 50/50 scenario was adopted for stability (WAASB) and yield to assigns equal importance, thereby avoiding bias toward either high-yielding but unstable genotypes or highly stable but low-yielding genotypes. This balanced approach is appropriate when the breeding objective is to identify genotypes that combine superior productivity with consistent performance across environments.

### Genotype Selection Index (GSI)

Each genotype’s modified GSI was computed using the computed indices rankings (ASI_R, WAAS_R, GGE_R, WAASB_R, WAASBY_R, and ER_R) in a single criterion (GSIi) as well as mean yield. [31,32]:


$$\begin{array}{c} GSIi\;=\;RYi\;+(RASIi+RWAASi+RGGEi+RWAASBi+RWAASBYi+RERi) \end{array}$$

where RYi is the rank of the ith genotype’s mean yield, GSIi is the genotype selection index for i^th^ genotype, RASIi, RWAASi, RGGEi, RWAASBi, RWAASBYi, and RERi are the ranks for ASI, WAAS, GGE, WAASB, WAASBY, and ER for the i^th^ genotype.

## Results

### Stability analysis

**Fig 2:**
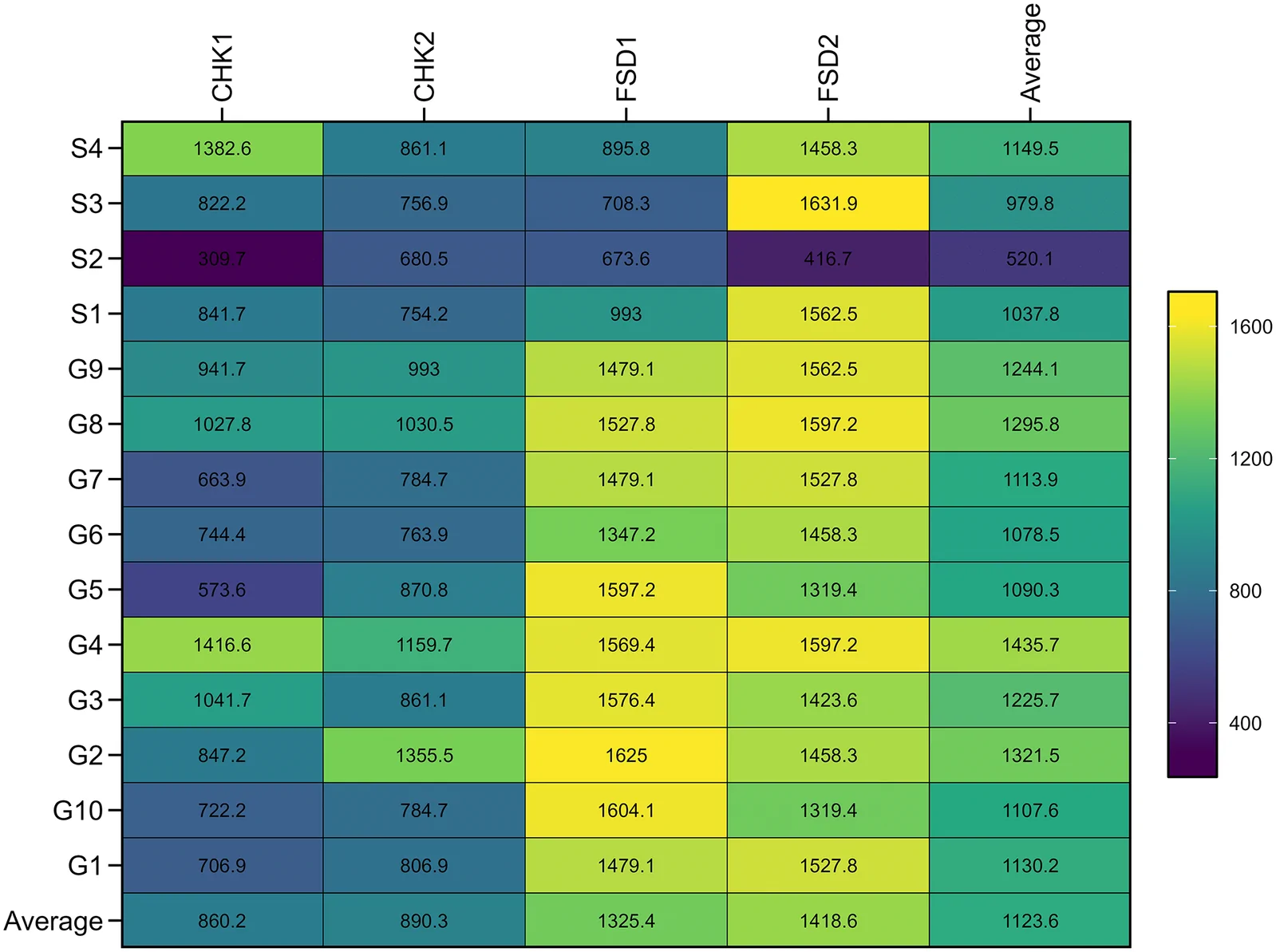
Heat map depicting genotypic and environmental mean (yield) of 14 lentil genotypes across four environments to visualize the variation in response across environment. FSD: Faisalabad; CHK, Chakwal with 1 & 2 indicates the years of study.

Pooled ANOVA results depicted presence of a significant genotype × environment interaction. Moreover, the main effects of genotypes and environments were also found significant (**S1 Table**). The variation in genotypes across the environment was dependent on the genotype and also on environment (Fig 2)

### Eberhart and Russell’s model

The Eberhart and Russell (1966) model was applied to assess the phenotypic stability of 14 genotypes (G1-G10 and S1-S4) across different environments. The analysis of variance (ANOVA) revealed that the genotypic effect (GEN) was highly significant (p = 0.0015), indicating substantial genetic variability among the genotypes evaluated (Table 2). This justified further examination of their individual stability parameters. The combined environmental and genotype × environment interaction effect [ENV + (GEN × ENV)] was also considerable, accounting for a large proportion of the total variation. Specifically, the linear environmental component (ENV linear) contributed the most to the interaction, suggesting that the environments were diverse and linearly affected genotype performance. However, the GEN × ENV (linear) component was non-significant (p = 0.401), indicating that differences among genotypes in response to environmental linear effects were not statistically meaningful.

**Table 2:** Eberhart and Russel’s ANOVA and pooled deviation.

Source	Df	Sum Sq	Mean Sq	F value	P value
Total	55	23642311.98	429860.22		
GEN	13	7051189.62	542399.20	3.80	0.00
ENV + (GEN x ENV)	42	16591122.36	395026.72		
ENV (linear)	1	10567376.86	10567376.86		
GEN x ENV (linear)	13	2031947.25	156303.63	1.10	0.40
Pooled deviation	28	3991798.25	142564.22		
G1	2	16919.00	8459.50	0.13	0.87
G10	2	257868.16	128934.08	2.05	0.13
G2	2	440971.47	220485.73	3.51	0.03
G3	2	163946.52	81973.26	1.30	0.28
G4	2	114088.54	57044.27	0.91	0.41
G5	2	352332.21	176166.11	2.80	0.07
G6	2	754.17	377.08	0.01	0.99
G7	2	22287.67	11143.84	0.18	0.84
G8	2	2678.11	1339.06	0.02	0.98
G9	2	1105.93	552.97	0.01	0.99
S1	2	360011.37	180005.69	2.86	0.06
S2	2	308741.60	154370.80	2.46	0.09
S3	2	1083395.71	541697.85	8.62	0.00
S4	2	866697.78	433348.89	6.89	0.00
Pooled error	104	6537495.61	62860.53		

**Fig 3:**
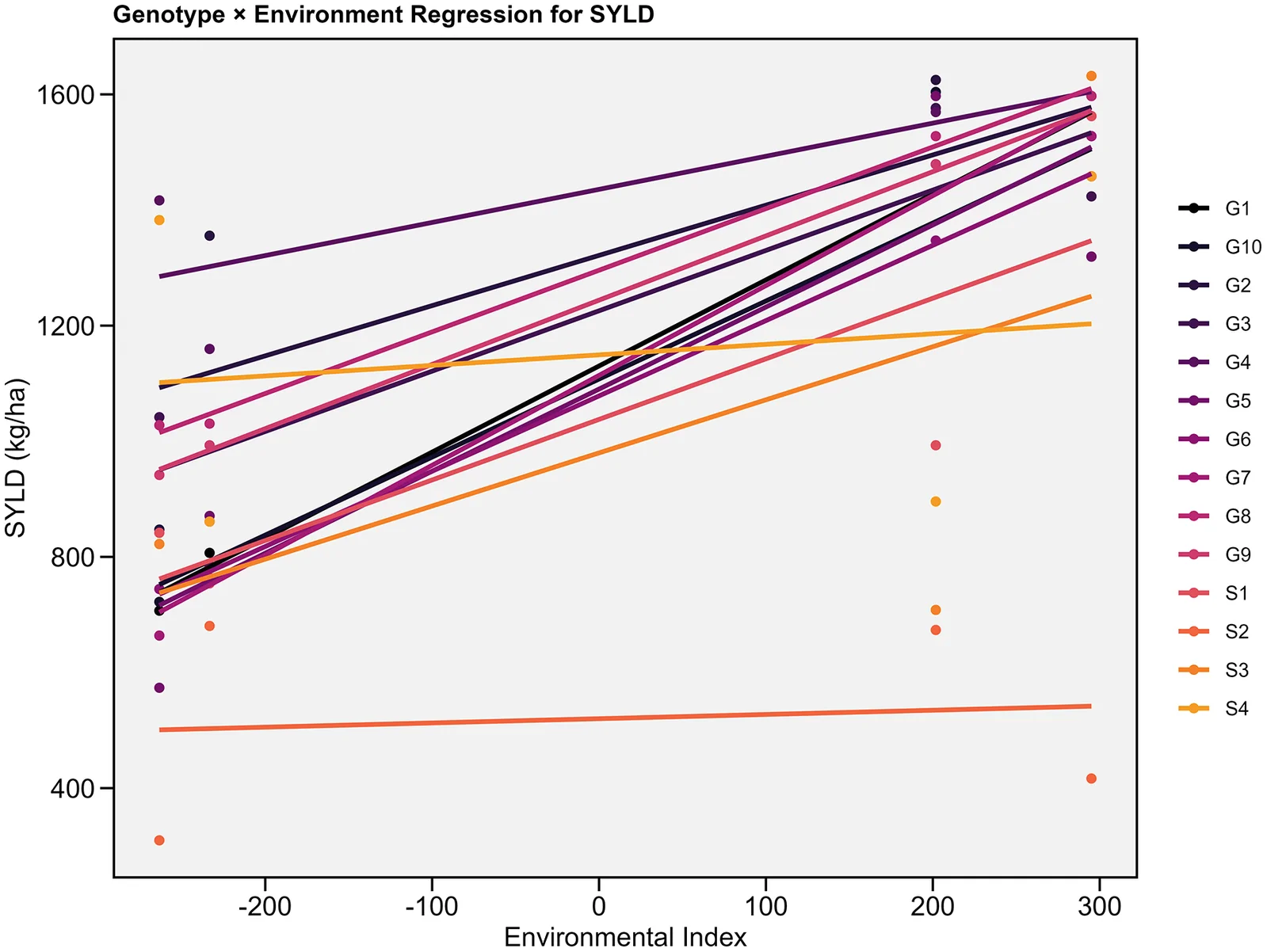
Regression plot of yield of 14 lentil genotypes across four environments to visualize the variation in response across environment.

The pooled deviation, representing the non-linear component of genotype × environment interaction, was significant, implying that genotypes differed in their non-linear response across environments and that simple linear regression may not be sufficient for all genotypes (Fig 3). The regression-based stability analysis was conducted using the Eberhart and Russell (1966) model to further evaluate genotype performance across environments. This approach estimated the mean performance (b₀), regression coefficient (b₁), deviation from regression (S^2^di), and associated statistical measures, including the coefficient of determination (R^2^) and root mean square error (RMSE). A genotype is considered stable and widely adapted if it exhibits mean yield > grand mean, a significant regression coefficient close to unity (b₁ ≈ 1), and a non-significant deviation from regression, indicating consistency across environments. Genotypes with high mean and significant b₁ > 1 with non-significant S^2^di are considered as below average in stability. Such genotypes are highly responsive to the change in environment and are less stable, hence, they perform fairly well in favorable environments but give poor yield in unfavorable conditions. Genotypes with significant b₁ < 1 with non-significant S^2^di and high mean are the least responsive ones and they do not respond favorably to better environmental conditions but, low response give these genotypes an edge to performs more stable in poor environmental conditions, so, their yield is not much effected (Eberhart and Russell 1966). Keeping in view these parameters genotypes were ranked based on their stability (responsiveness and suitability for environment type) and yield, finally a cumulative rank was calculated (Table 3).

**Table 3:** Eberhart and Russel’s Regression analysis and ranking of genotypes based on yield and stability.

GEN	b_0_ (mean)	b_1_	t-test (p values)	Responsiveness (based on b_1)_	S^2^di	f-test (p values)	Stability (based on S^2^di)	Suitability	RMSE	R2	Stability rank	Yield rank	Cumulative score	Final Rank
**(response based)**
G1	1130	1.49	0.09	Average	−18133.68	0.87	Stable	Favourable	37.55	0.99	1	7	8	6
G10	1108	1.35	0.23	Average	22024.52	0.13	Stable	Favourable	146.59	0.84	1	9	10	9
G2	1322	0.87	0.65	Average	52541.73	0.03	Unstable	Favourable	191.70	0.56	3	2	5	3
G3	1226	1.04	0.88	Average	6370.91	0.28	Stable	Favourable	116.89	0.83	1	5	6	5
G4	1436	0.57	0.14	Average	−1938.75	0.41	Stable	Favourable	97.51	0.68	1	1	2	1
G5	1090	1.42	0.15	Average	37768.52	0.07	Stable	Favourable	171.35	0.81	1	10	11	10
G6	1078	1.30	0.30	Average	−20827.82	0.99	Stable	Favourable	7.93	1.00	1	11	12	11
G7	1114	1.55	0.06	Average	−17238.90	0.84	Stable	Favourable	43.10	0.99	1	8	9	7
G8	1296	1.07	0.82	Average	−20507.16	0.98	Stable	Favourable	14.94	1.00	1	3	4	2
G9	1244	1.11	0.70	Average	−20769.19	0.99	Stable	Favourable	9.60	1.00	1	4	5	3
S1	1038	1.05	0.87	Average	39048.38	0.06	Stable	Favourable	173.21	0.70	1	12	13	12
S2	520	0.07	0.00	Low	30503.42	0.09	Stable	Unfavourable	160.40	0.01	1	14	15	13
S3	980	0.92	0.78	Average	159612.44	0.00	Unstable	Favourable	300.47	0.37	3	13	16	14
S4	1149	0.18	0.01	Low	123496.12	0.00	Unstable	Unfavourable	268.75	0.03	3	6	9	7

GEN; Genotypes, RMSE; Root Mean Square Error

All the test entries showed average response (b₁ values and significance), with only 02 check varieties S2 and S4 being low responsive. However, their response to environment (non-significant deviation from S^2^di) was stable for most of the genotypes except G2. As for yield, among the test entries, G4 was the top performer with 1436 kg/ha yield which was also> than grand mean (1124 kg/ha), while G6 was lowest (1078 kg/ha). Overall ranking showed that G4 was the top ranked with highest yield and stable response, followed by G8, G2, G9, G3, G1 and G7. G10, G5 and G6 had mean lower than grand mean and were ranked last. Among the top 07 lines, yield was higher than grand mean and also from checks, however their stability played role. Both G2 and G9 ranked 3^rd^, however, G2 was unstable and can be recommended for favorable stable environments only.

### AMMI analysis

**Fig 4:**
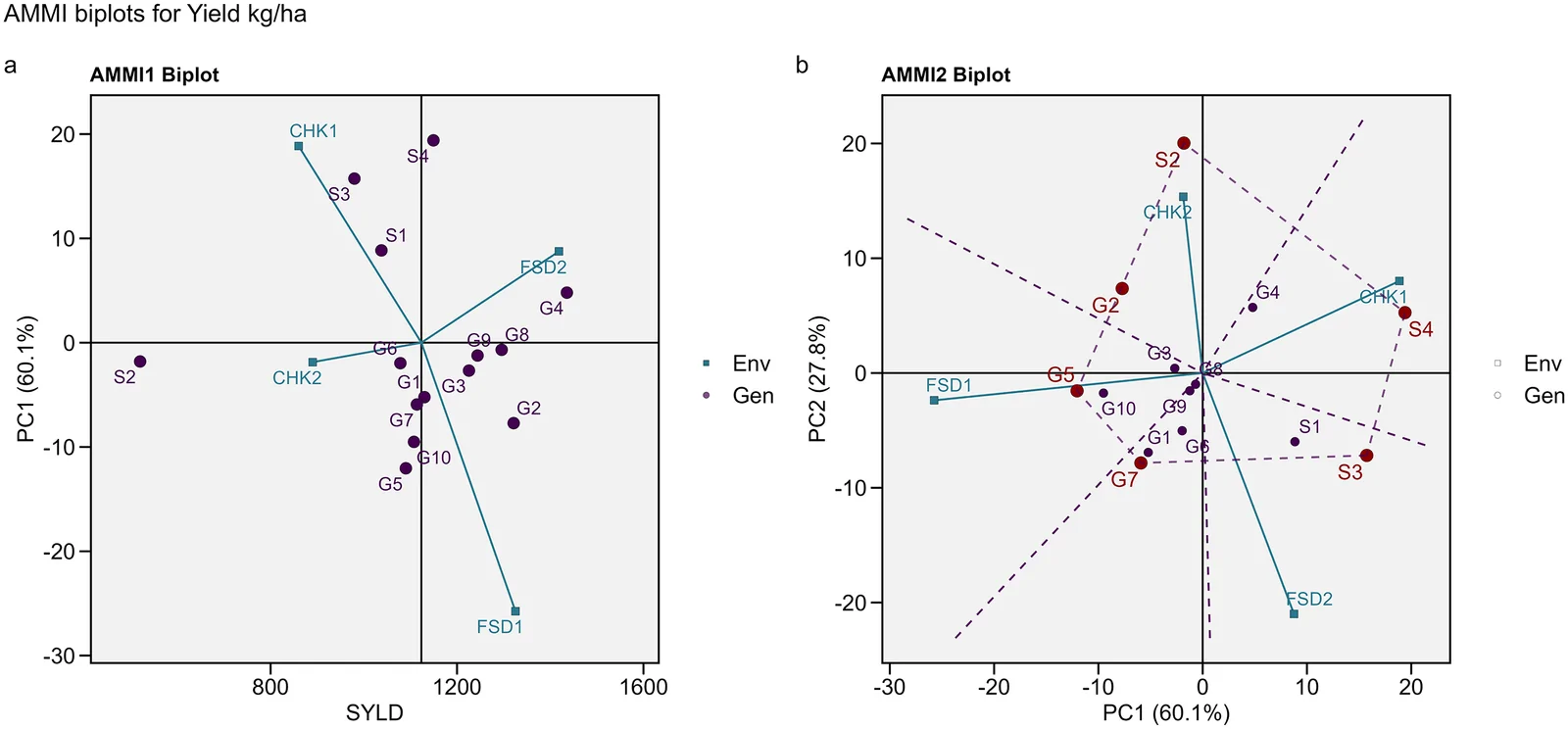
a) AMMI1 biplot between mean yield (kg ha^−1^) and PC1 representing the distribution of environments and genotypes on basis of interaction and additive effects. b) AMMI2; PC1 and PC2 plotted to get a polygon view, the genotypes in the centre of the polygon are the stable one. SYLD, seed yield; FSD, Faisalabad; CHK, Chakwal; PC, principal component.

Genotype (G), environment (locations × years), and their interaction (G × E) all significantly influenced the yield of lentil genotypes, according to the AMMI ANOVA result (p < 0.001) (Table 4). Environment accounted for the largest amount of variance in yield, with the highest explained sum of squares value (28.5%), followed by genotype (19.0%) and G × E (16.2%). Cumulatively the main and interaction effect explains 63.7% of model. PCA was performed on the G × E interaction matrix obtained from the ANOVA. It decomposed the interaction into a three interaction principal component axes (IPCA). The first two IPCs significantly explained 60% and 27.8% of the interaction variation, respectively, together accounting for 87.8% of the total 14.3% variation attributed to genotype × environment interaction. Based on analysis results two bi-plots were generated: AMMI1 and AMMI2. In AMMI1 bi-plot, mean (yield) is on x-axis and IPCA1 is on y-axis. These axes are orthogonal and divide the plot area into four quadrates [1]. Class I includes high-yielding genotypes with strong positive interaction, while Class II combines high yield with weak negative interaction. Class III represents low yield with minimal interaction, and Class IV includes low-yielding genotypes with strong interaction. The vertical line indicates the grand mean value (1124 kgha^-1^), so all the genotypes on the right are high yielding. Distance from origin indicates the stability status of genotypes; the closer a genotype is to the origin more stable they are with low G × E contribution compared to those farther from the origin. 07 genotypes (including check S4) showed mean yield higher than grand mean, however the order of distance from origin G9 > G3 > G1 > G8 > G2 > G4 > S4 depicts that G9 was the most stable one while S4 being the least stable (Fig 4a).

**Table 4:** AMMI ANOVA of yield (kg ha − 1) of 14 lentil genotypes at two locations in Pakistan during two growing seasons (2022-23 & 2023-24).

Source	Df	Sum Sq	Mean Sq	F value	Proportion	Accumulated	Explained%
ENV	3	10567376.86	3522458.95	30.60***			28.5
REP(ENV)	8	920871.04	115108.88	1.83			2.5
GEN	13	7051189.62	542399.20	8.63***			19.0
GEN:ENV	39	6023745.50	154455.01	2.46***			16.2
PC1	15	3622453.02	241496.87	3.84***	60.10	60.10	9.8
PC2	13	1672510.30	128654.64	2.05**	27.80	87.90	4.5
PC3	11	728782.17	66252.92	1.05	12.10	100.00	2.0
Residuals	104	6537495.615	62860.53476				82.4
Total	206	37124424.12	180215.6511				

* = significant at p < 0.05; **: significant at p < 0.01; ***: highly significant at p < 0.001. Df degree of freedom; Sq square; ENV environment; REP replication; GEN genotypes; PC principal component

The AMMI2 bi-plot (IPCA1 vs. IPCA2) captures the first two IPCs and effectively represents the G × E pattern. Genotypes with high PC1 scores and low absolute PC2 values are considered ideal, whereas those near origin are viewed as stable. In AMMI2, a polygon is drawn to see which of the genotypes are stable, moreover, the dotted lines divide the plot into sectors. The environment and genotypes sharing same sector are considered compatible. G2, G5, G7, S2, S3 and S1 located at the vertices of the polygon, have the longest vectors and are considered the least stable due to their distance from the origin. G8, G9 and G3 are the most stable genotypes as they are close to the origin. Among the environments, CHK1 and FSD2 had the longest vectors suggesting their high contribution to interaction (Fig 4b). AMMI model-based indices (ASI and WAAS) were calculated to facilitate the genotypes ranking (Table 5).

**Table 5:** Ranking of genotypes according to AMMI model-based stability indices (ASI and WAAS).

GEN	Y	Y_R	ASI	ASI_R	WAAS	WAAS_R
G1	1130.19	7	4.17	7	6.53	7
G10	1107.62	9	5.67	10	7.57	9
G2	1321.51	2	0.86	2	1.33	2
G3	1225.67	5	3.29	5	5.09	5
G4	1435.74	1	0.49	1	0.78	1
G5	1090.26	10	5.07	8	7.60	10
G6	1078.45	11	5.57	9	7.94	11
G7	1113.87	8	5.74	11	7.06	8
G8	1295.81	3	1.61	3	1.96	3
G9	1244.08	4	1.83	4	2.93	4
S1	1037.83	12	7.25	12	8.73	12
S2	520.13	14	11.75	14	14.93	14
S3	979.85	13	9.66	13	13.03	13
S4	1149.46	6	3.68	6	5.76	6

GEN; Genotypes, Y; Yield, Y_R; Yield Rank, ASI; AMMI Stability Index, ASI_R; ASI Rank, WAAS; Weighted Average of Absolute Scores, WAAS_R; WAAS Rank.

### GGE-Biplots

The Average Environment Coordinate (AEC) system was used to assess the mean yield performance and stability of genotypes. In this system, PC1 (x-axis) represents mean yield, while PC2 (y-axis), perpendicular to PC1, reflects genotype stability. GGE bi-plot analysis showed that PC1 and PC2 explained 56.94% and 27.58% of the variation, respectively, accounting for a total of 84.52%.

**Fig 5:**
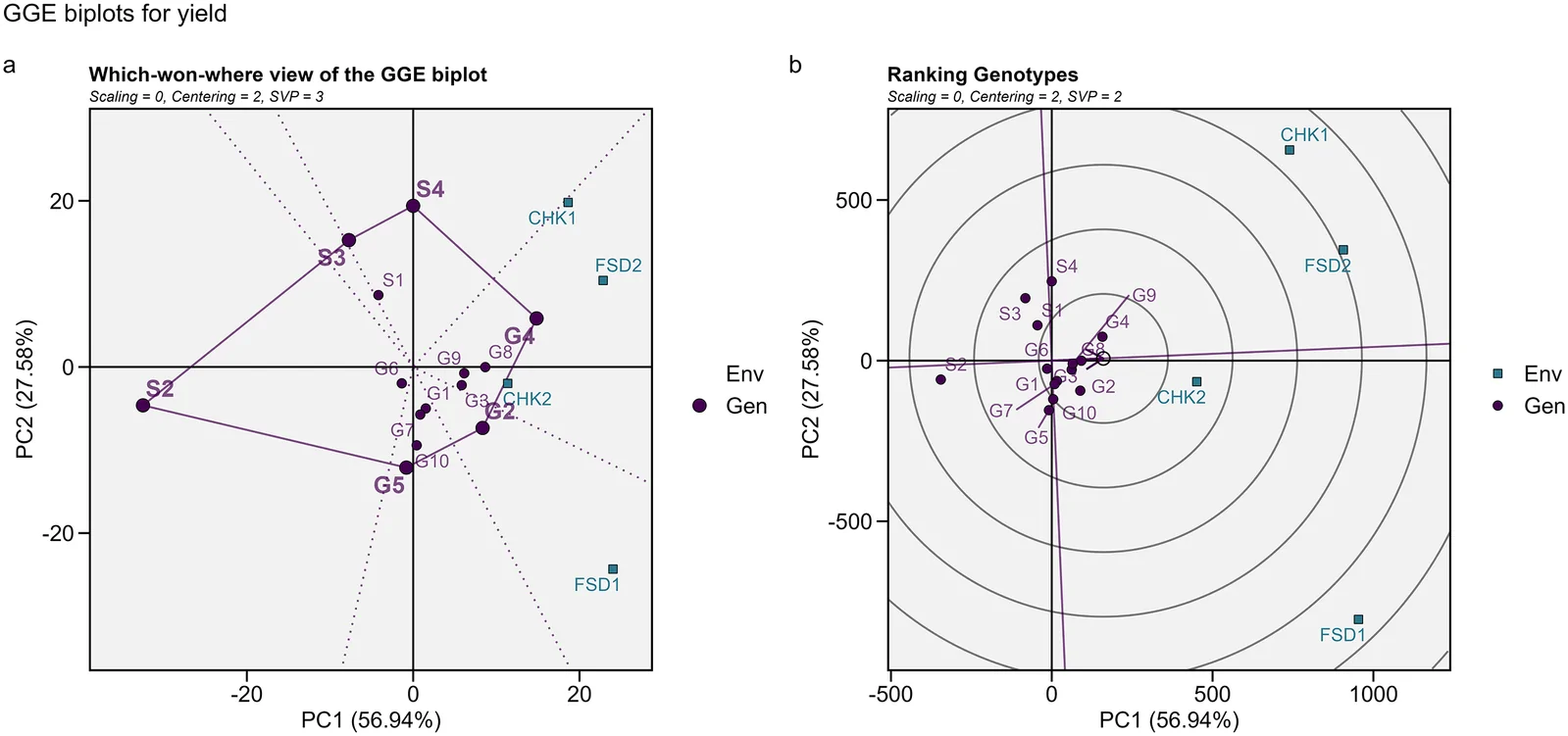
a) Which-won-where GGE biplot showing 14 genotypes and 04 environments, the genotypes sharing same sector with an environment are the most suitable genotypes for that specific environment in the same sector b) Ranking of genotypes view of GGE biplot on basis of a hypothetical ideal genotype in the centre of concentric circles. SYLD, Seed yield; PC, principal component; FSD, Faisalabad; CHK, Chakwal.

The which-won-where GGE bi-plot, as the name indicates, describes the genotypes and environments selectivity towards each other (Fig 5a). This plot also explains the G × E mode (cross-over or non-cross-over) through the polygon view, which is similar to AMMI2 bi-plot. Genotypes sharing same sector with a particular environment are considered suitable for that environment. However, in contrast to AMMI2, the genotypes present at vertex of polygon are considered the winning genotypes for the sector. CHK1, CHK2 and FSD2 shared sector with G4, G8, G9 and G3, with G4 being the winning genotype for this sector. FSD1 shared sector with G2, indicating its high performance in this environment. In this plot, genotypes S2, S3, S4, G4, G5 and G2 are positioned at the vertices of the polygon, indicating that they performed best in at least one environment. These genotypes are highly responsive and potentially less stable due to their distance from the origin. Conversely, genotypes located near the origin, such as G6, G1, G8, and G9 are more stable and less sensitive to environmental changes. Some genotypes do not share sector with any of the environments and are present in a contagious region, the interaction will be more complex than simple positive and negative interactions.

In the GGE bi-plot (ranking genotypes), the centre of the concentric circles with a small arrow represents the hypothetical ideal genotype, serving as a reference point (Fig 5b) (Yan & Tinker, 2006). The genotype closest to this centre is considered the most desirable. In this case, G4 is closest to the ideal genotype, making it the most preferred. The genotypes can be ranked based on their distance from ideal as: G4 > G2 > G8 > G9 > G3 > G1 > G7 > G10 > S4 > G5 > G6 > S1 > S3 > S2. The environments FSD1, FSD2 and CHK1 have the longest vectors, indicating high discriminating ability and strong influence on genotype differentiation. In contrast, CHK2 lies closer to the center, showing moderate discriminating ability. Overall, the plots confirm the presence of crossover G × E interactions, as different genotypes perform best in different environments.

### WAASB & WAASBY (based on BLUPs)

**Fig 6:**
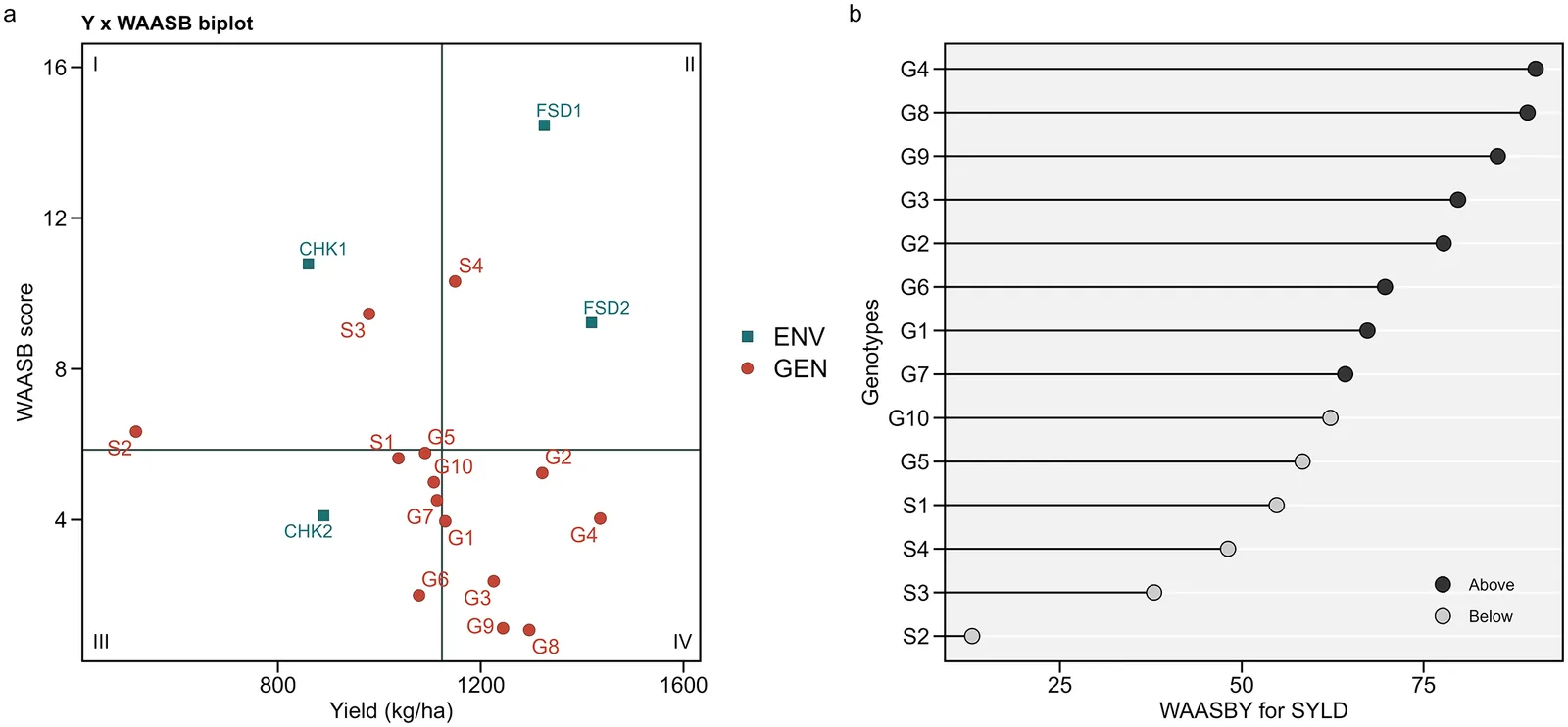
a) Y × WAASB biplot showing distribution of 14 genotypes across 04 environments. This biplot depicts the G × E interaction on basis of yield and stability (WAASB) b) Ranking of genotypes based on their WAASBY index with equal weights of yield and stability. SYLD, Seed yield; FSD, Faisalabad; CHK, Chakwal; WAASB, The Weighted Average of Absolute Scores for BLUPs; WAASBY, Weighted Average of Absolute Scores and Yield.

WAASB values were premeditated based on BLUPs, it assigns a stability score to the genotype, the lower the score, the more stable the genotype will be. The Yield × WAASB bi-plot was generated with SYLD on x-axis and WAASB score on y-axis (Fig 6a). This bi-plot visually assesses the performance and stability of genotypes across environments. An ideal genotype would have a high yield (right side of the plot) and a low WAASB score (bottom of the plot), indicating both productivity and stability. The plot is divided into four quadrants: Quadrant IV (bottom-right) includes genotypes that exhibit high yield and high stability and are thus the most desirable for selection. Genotypes such as G4, G2, G8, G9, G3, and G1 fall in this quadrant, with G4 particularly standing out for its superior yield and good stability. Quadrant II (top-right) represents genotypes with high yield but poor stability, including S4, FSD1, and FSD2, which may still be valuable in favorable or managed environments. Quadrant III (bottom-left) includes stable but low-yielding genotypes like G7, G10, G5, G6 and S1 and CHK2. Quadrant I (top-left) includes genotypes with both low yield and low stability, such as CHK1, making them the least favorable choices. Overall, the bi-plot highlights genotypes in Quadrant IV, especially G4, as promising candidates for recommendation based on their combined high yield and stability. The WAASBY index was calculated for all genotypes, keeping equal weights for yield and stability (WAASB) and ranks were determined (Table 6).

**Table 6:** Ranking of genotypes according to BLUPs based WAASBY index.

GEN	Y	Y_R	WAASB	WAASB_R	WAASBY	Mean	WAASBY_R
G1	1130.19	7	3.96	5	67.72	Above	7
G10	1107.62	9	5.00	8	60.88	Below	9
G2	1321.51	2	5.24	9	71.24	Above	6
G3	1225.67	5	2.37	4	81.53	Above	4
G4	1435.74	1	4.03	6	84.01	Above	3
G5	1090.26	10	5.77	11	55.75	Below	10
G6	1078.45	11	2.00	3	75.51	Above	5
G7	1113.87	8	4.52	7	63.81	Above	8
G8	1295.81	3	1.08	1	92.36	Above	1
G9	1244.08	4	1.13	2	89.27	Above	2
S1	1037.83	12	5.63	10	53.63	Below	11
S2	520.13	14	6.34	12	21.55	Below	14
S3	979.85	13	9.46	13	29.76	Below	13
S4	1149.46	6	10.32	14	34.37	Below	12

GEN; Genotypes, Y; Yield, Y_R; Yield Rank, WAAS; Weighted Average of Absolute Scores, WAAS_R; WAAS Rank, WAASBY; Weighted Average of Absolute Scores and Yield

The WAASBY index gives a single value for each genotype, higher value is desirable. It assigns a cut-off value and rank genotypes above (good) and below (poor) that cut-off. The index plot is generated for yield ranks genotypes based on their weighted average scores (Fig 6b). In this plot, genotypes are arranged along the y-axis, with their corresponding WAASBY values displayed on the x-axis. Dark-filled points indicate genotypes that performed above the average WAASBY index, whereas light-filled points represent those below the average. Genotypes such as G4, G8, G9, G3, and G2 are positioned at the top of the plot with the highest WAASBY values, indicating superior performance in both yield and stability. G4 in particular stands out as the top-ranking genotype. On the other hand, genotypes like S2, S3, S4, and S1, which appear at the bottom and have lower WAASBY scores, showed suboptimal performance and stability. This plot confirms that genotypes with high WAASBY indices, especially those in the top half such as G4, G8, and G9, are ideal candidates for selection due to their desirable combination of productivity and adaptability across environments.

### Genotype Selection Index (GSI) & Genetic Parameters

**Fig 7:**
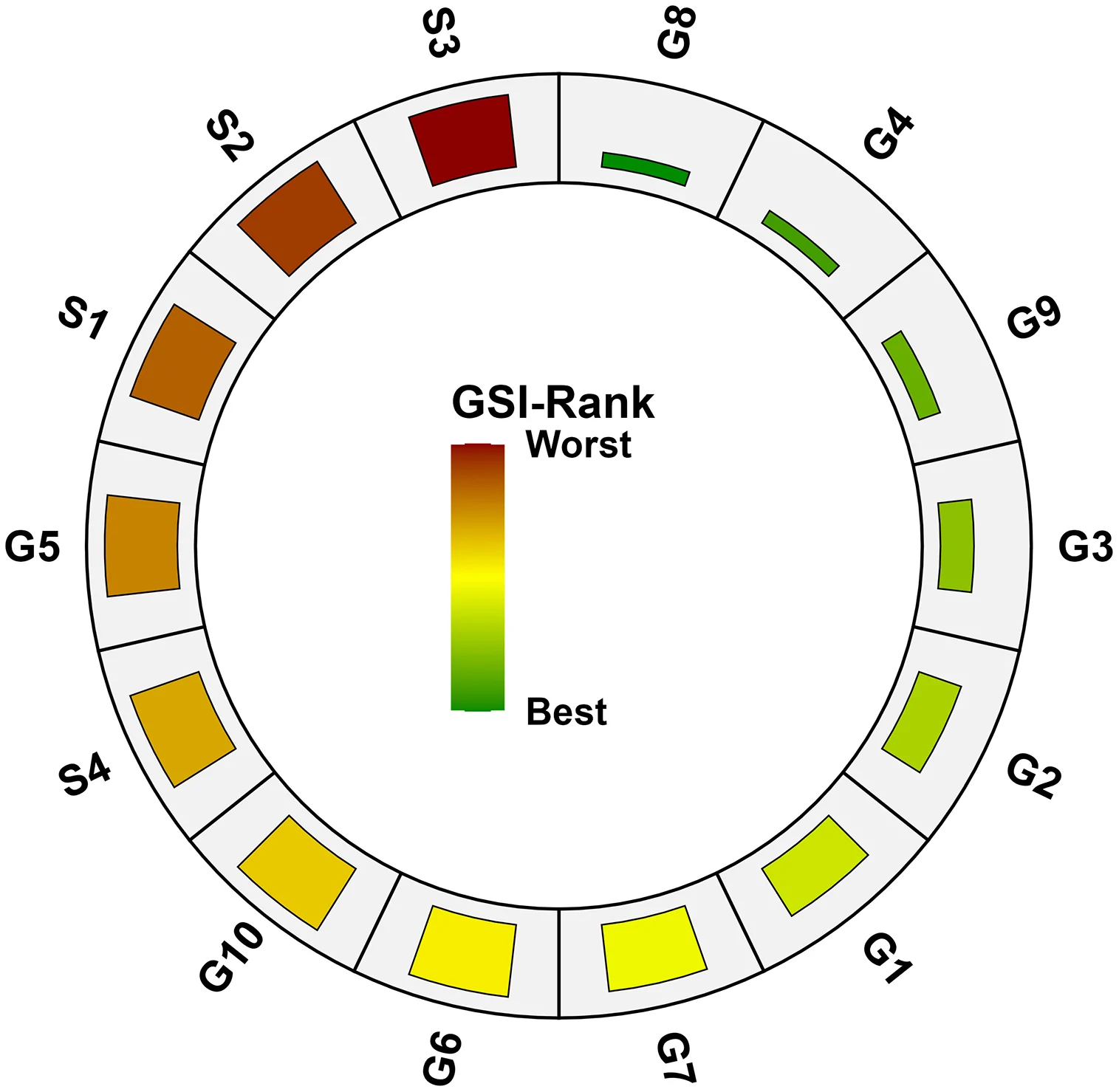
Genotypes Selection Index (GSI) of 14 genotypes based on 06 stability ranks (Y, Yield; ASI, AMMI-Stability Index; WAAS, Weighted Average of Absolute Scores from AMMI; GGE, WAASBY, WAASB + Yield Index) using the cumulative rank score, lowest rank being the best selected genotypes.

GSI was calculated as a cumulative rank score for a comprehensive evaluation of genotypic performance based on multiple stability and yield indices to identify the most productive and stable genotypes (Table 7). Each genotype (Codes: G1 to G10, and S1 to S4) was assessed using several criteria: yield rank (Y_R), AMMI Stability Index rank (ASI_R), WAAS stability rank (WAAS_R), GGE bi-plot stability rank (GGE_R), WAASBY index rank (WAASBY_R), and Eberhart-Russell Rank (ER_R). The cumulative index GSI combines all these ranks to provide an integrated performance score, where a lower GSI indicates better performance. GSI_R is the final ranking of the genotypes based on their GSI scores (Fig 7).

**Table 7:** Genotypes Selection Index (GSI) calculated from indices determined from multiple stability models and the genetic parameters for selection.

GEN	Mean ± SE	Y_R	ASI_R	WAAS_R	GGE_R	WAASBY_R	ER_R	GSI	GSI_R
G1	1130.19 ± 120.85 b-f	7	6	6	6	7	6	38	6
G10	1107.62 ± 118.62 c-f	9	11	8	8	9	9	54	9
G2	1321.51 ± 126.05 ab	2	8	10	3	6	3	32	5
G3	1225.67 ± 115.74 b-e	5	3	3	5	4	5	25	4
G4	1435.74 ± 78.66 a	1	5	5	1	3	1	16	2
G5	1090.26 ± 125.69 def	10	12	12	10	10	10	64	11
G6	1078.45 ± 144.66 def	11	4	4	11	5	11	46	8
G7	1113.87 ± 132.81 c-f	8	7	7	7	8	7	44	7
G8	1295.81 ± 119.06 abc	3	1	1	2	1	2	10	1
G9	1244.08 ± 93.17 a-d	4	2	2	4	2	3	17	3
S1	1037.83 ± 115.09 ef	12	9	11	12	11	12	67	12
S2	520.13 ± 52.55 g	14	10	9	14	14	13	74	13
S3	979.85 ± 118.24 f	13	13	13	13	13	14	79	14
S4	1149.46 ± 104.12 b-f	6	14	14	9	12	7	62	10
gMean	1123.61 ± 72.30	ECV (%)	22.30						
LSD at 5%	202.89	GCV (%)	31.13						
LSD at 1%	269.51	GCV (%)	38.30						
σ²ₑ	62860.53	H² (bs)	0.66						
σ²g	122365.65	GA	585.85						
σ²p	185226.18	GAM (%)	52.14						

Note: gMean; Grand Mean, LSD; Least Significant Difference, σ²ₑ; Environmental Variance, σ²g; Genotypic Variance, σ²p; Phenotypic Variance, ECV (%); Environmental Coefficient of Variance, GCV (%); Genotypic Coefficient of Variance, PCV (%); Phenotypic Coefficient of Variance, H² (bs); Heritability (Broad Sense), GA; Genetic Advance, GAM (%); Genetic Advance as percentage of mean.

Results showed that G4 stands out as the top-performing genotype with the highest mean yield (1436 kg ha ⁻ ¹) and an overall GSI of 16, ranking it second (GSI_R = 2) among all entries. However, G8 is the best overall performer (GSI_R = 1) with a GSI of 10, due to its consistently top rankings across all stability indices: it ranked 3rd in yield (Y_R = 3), 1st in ASI_R, WAAS_R, and WAASBY_R, and 2nd in GGE_R and ER_R. G9 also showed excellent performance, ranking 3rd in GSI_R with good scores across yield and stability metrics. Genotypes G3 and G2 followed closely, with GSI_R values of 4 and 5, indicating balanced performance. On the other hand, genotypes like S3, S2, and S1 performed poorly both in yield and stability, with the highest GSI values (79, 74, and 67 respectively), placing them at the bottom of the ranking (GSI_R = 14, 13, and 12, respectively). These genotypes are less desirable due to their low productivity and poor adaptability. Intermediate performers like G6, G7, and G10 had moderate yields and stability, ranking 8th, 7th, and 9th respectively. In conclusion, G8, G4, and G9 are the most promising genotypes for selection due to their superior yield and overall stability across environments, as indicated by their low GSI scores and top ranks.

Genetic parameters were also calculated to get a deeper view of the variability among the tested genotypes. A large portion of the observed variation was genetically regulated, as indicated by the genotypic variance (σ²g = 122,365.65) being greater than the environmental variance (σ²ₑ = 62,860.53). Success of selection was depicted by the broad-sense heritability (H2 = 0.66) which indicates a reasonably strong level of genetic control over the characteristic. This finding was further supported by the presence of additive gene action as indicated through the genetic advance (GA = 585.85) and genetic advance as percentage of mean (GAM = 52.14%). The phenotypic coefficient of variance (PCV = 38.30%) was slightly higher than the genotypic coefficient of variance (GCV = 31.13%), indicating that genetic factors predominated, but environmental factors also had some influence. Overall, these metrics show that the trait under study had significant genetic variability, which makes it suitable for efficient selection in lentil breeding programme.

### Spearman’s Rank correlation

**Fig 8:**
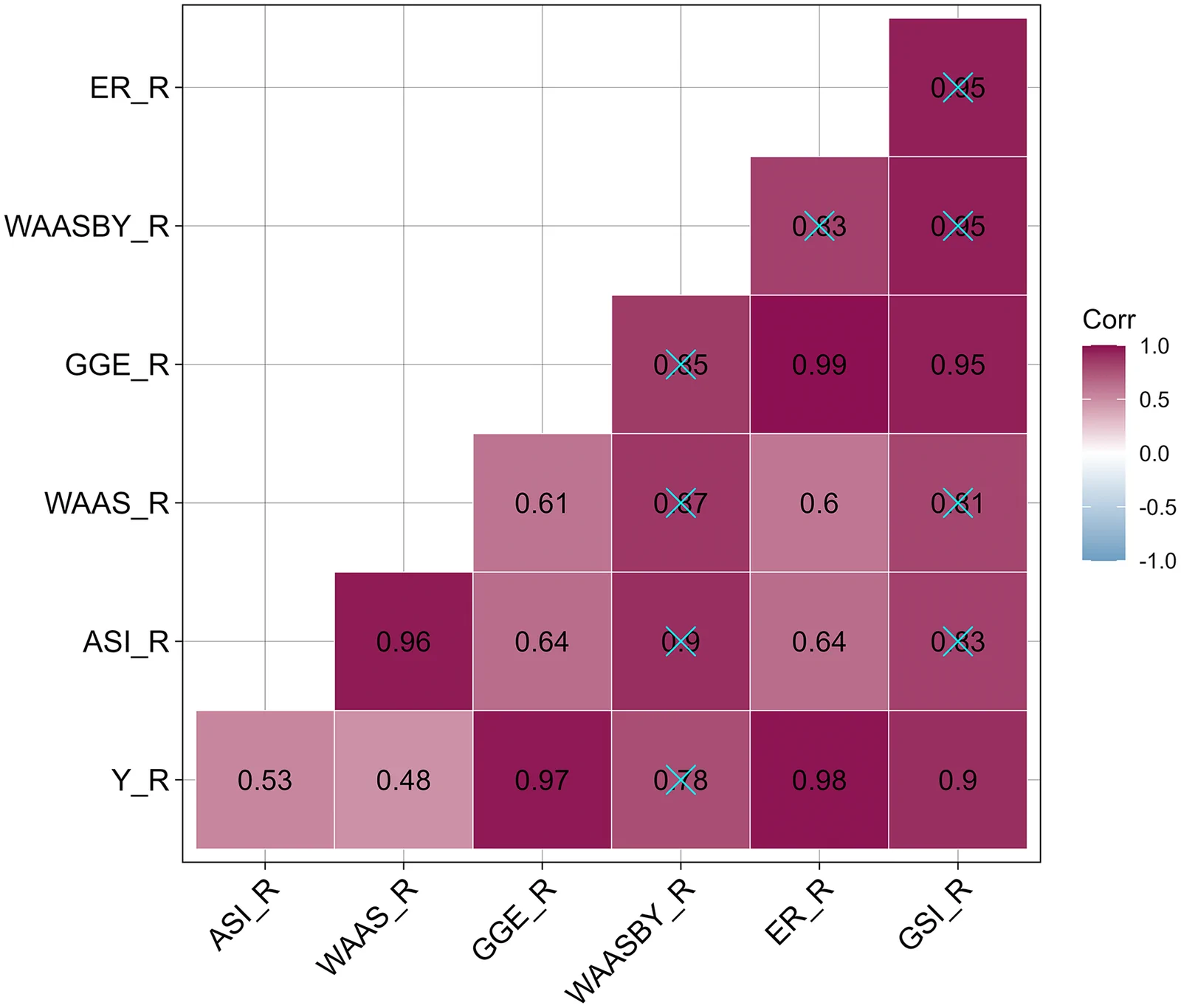
Spearman’s Rank correlation between ranks of all stability indices. The cross on the cells depicts non-significance at p < 0.05. Y_R, Yield_Rank; ASI, AMMI-Stability Index; ASI_R, AMMI-Stability Index_Rank; WAAS, Weighted Average of Absolute Scores from AMMI; WAAS_R, Weighted Average of Absolute Scores_Rank; GGE_R, GGE; WAASBY_R, WAASB + Yield Index_Rank; GSI_R, Genotype selection index rank; ER_R, Eberhart-Russell Ranks.

Spearman’s rank correlation analysis revealed strong interrelationships among the various yield and stability indices used to evaluate genotype performance (Fig 8). Notably, yield rank (Y_R) showed a very strong and significant correlation with Eberhart-Russell rank (ER_R) (r = 0.98, p < 0.001) and GGE rank (GGE_R) (r = 0.97, p < 0.001), highlighting the strong influence of environmental factors and GGE analysis on yield performance. The WAASBY rank, an integrative index combining yield and stability, demonstrated strong correlations with ASI_R (r = 0.90), WAAS_R (r = 0.87), and GSI_R (r = 0.95), supporting its robustness as a selection tool. The GSI_R, which aggregates all indices into a comprehensive selection metric, showed consistently high correlations with major individual indices such as GGE_R (r = 0.95, p = 0.014), ER_R (r = 0.95, p = 0.018), and WAASBY_R (r = 0.95, though p = 0.568), affirming its effectiveness in identifying superior genotypes. While ASI_R had moderate correlations with Y_R and stronger association with WAAS_R (r = 0.96), it contributed useful supplementary insights. Although some high correlation coefficients were statistically non-significant, likely due to the limited number of genotypes and tied ranks, the overall analysis underscores the reliability of GSI and WAASBY indices in integrating yield and stability for genotype selection.

## Discussion

A crucial phenomenon in agricultural genetics, the genotype-environment (G × E) interaction explains how different genotypes function in various environmental settings. Plant breeders that are interested in creating novel cultivars which thrive consistently in a variety of conditions must comprehend and measure G × E interactions. However, selection efficiency in breeding programs can be reduced by interaction, which makes it more difficult to choose superior genotypes because of the varying performance of varieties in relation to environmental conditions [33]. This demonstrates that in order to make well-informed breeding decisions, particular analytical models that can accurately describe the underlying dynamics of this interaction are required. In this study, multiple stability models, including Eberhart and Russell regression model, AMMI, GGE, and BLUP based indices WAAS and WAASBY were applied to evaluate lentil genotypes across environments.

In our study, the pooled ANOVA confirmed significant effects of genotype, environment, and their interaction, highlighting the complexity of lentil yield performance across variable conditions. Environmental factors explained the largest share of variation, emphasizing the importance of stability analysis in identifying cultivars that combine high yield with resilience under diverse environments. Genotypes showed high variation in mean yield values and ranking based on yield, b and S^2^di. The regression model of Eberhart and Russell [34] provided insights into genotype responsiveness, where all lentil genotypes showed average responsiveness (b₁ ≈ 1) and non-significant deviations, except for G2, indicating stability. G4 was the highest-yielding and most stable genotype (1436 kg ha ⁻ ¹), followed by G8 and G9. Although G2 ranked similarly to G9, its instability suggests adaptation mainly to favourable environments, whereas G9 showed broader adaptability. G10, G5, and G6 were less promising due to yields below the grand mean. Significant pooled deviations revealed that several genotypes exhibited non-linear responses, which this model could not fully capture. It oversimplifies stability by classifying genotypes into broad categories, assumes linear responses that may not reflect complex genotype × environment interactions, and often explains only a small portion of variability [35]. Sharifi et al. [36] proposed that the variation in mean yield of genotypes across the locations may be due to variation in sowing date, soil types, amount of rainfall, humidity, and sunshine hours during the crop life cycle. This method also struggles with unbalanced data, heterogeneous variances, and non-orthogonal designs, and it treats genetic effects as fixed, limiting analytical flexibility [37]. Moreover, it requires a sufficient number of replicated environments to yield reliable estimates. Thus, although useful for basic stability assessment, it lacks the depth and robustness needed for modern multi-environment trials [34]. Its reliance on linear assumptions limits its ability to reflect complex genotype × environment (G × E) interactions [15].

The AMMI model effectively partitioned additive and interaction effects, identifying G9, G3, and G8 as stable genotypes, while G2 and G5 showed environment-specific adaptability. The highest value of explained sum of squares was credited to the environment (28.5%), followed by genotype (19.0%) and G × E interaction (16.2%) which shows that the main cause of variation in seed yield was the environment factor. This information is very helpful because it explains 63.7% of the model. The strength of AMMI is its ability to separate the additive effects of environments and genotypes from the multiplicative G × E interaction, which reveals the extent and patterns of interaction and genotype responses across environments [38]. Moreover, ASI was also utilized to capture information from AMMI. Unlike simple variance measures, ASI captures the complexity of interaction patterns and, when combined with mean yield, helps identify genotypes that are both high-yielding and stable [39,40]. However, AMMI1 bi-plots provide limited information about discriminating environments, while higher-order components in AMMI2 may complicate interpretation and risk over-fitting unless carefully applied [41,42]. This limitation can be eased by combining the insights of the AMMI model with BLUP to generate the WAASB index, which captures genotype stability by taking into account all interaction main components, not only the first one [24].

The GGE bi-plot captured over 84% of the variation, effectively revealing “which-won-where” patterns. This model is particularly effective for visualizing crossover interactions and delineating mega-environments [22,43]. The “which-won-where” bi-plot revealed clear crossover G × E interactions, with G4 being the winning genotype for CHK1, CHK2, and FSD2, while G2 performed best in FSD1. G4 was also closest to the ideal genotype, followed by G2, G8, and G9, indicating their superior overall performance and adaptability. FSD1, FSD2, and CHK1 showed high discriminating ability, as compared to CHK2. Overall, the results confirmed that genotype performance varied across environments, highlighting the importance of multi-environment testing for identifying widely adapted genotypes. The identification of desirable genotypes with relation to their relative distance from the ideal genotype in GGE bi-plot were also reported in other legumes stability studies; lentil [1], mungbean [44], urdbean [45] and chickpea [46]. Nonetheless, GGE bi-plots may capture only a small proportion of the total G + GE variation when genotype main effects are small or when interaction structures are highly complex, thereby limiting its predictive accuracy. Moreover, it is sensitive to outliers and may be less flexible for missing data and heterogeneous variances. Therefore, GGE bi-plot results should be interpreted cautiously and supported by complementary statistical methods [23].

BLUP-based methods, particularly WAASB and WAASBY, provided a more integrated and breeder-oriented perspective [47]. WAASB summarize stability across multiple interaction principal component axes (IPCs), while WAASBY combine yield and stability into a single index, facilitating straightforward selection [48]. In this study, WAASBY consistently ranked G4, G8, and G9 among the best-performing genotypes, in agreement with other models, but with greater simplicity and interpretability. Recent studies have confirmed the robustness of WAASBY in different crops, including maize [49], sorghum [50], wheat [51], and rice [52], where it proved effective in identifying high-yielding and stable genotypes across multi-environment trials. Furthermore, WAASBY outperforms traditional indices when balancing yield stability and genetic gain in several breeding programs. However, this also counts as its limitation, because the final ranking depends on the weights assigned to performance and stability, so results may change according to breeder preference, and it still requires well-designed multi-environment data for reliable interpretation [24].

Despite the advantages, no single model is universally reliable, because each method has its own limitations too. Regression models provide insights into responsiveness, AMMI reveals detailed G × E structures, and GGE aids mega-environment identification. Their individual limitations highlight the value of applying multiple models, ensuring cross-validation and a holistic understanding of genotype adaptability [53]. However, our findings demonstrated that WAASBY stands out as an efficient tool that can also be applied independently, especially in resource-limited or time-constrained breeding programs where quick yet reliable decisions are necessary [54]. It identifies superior genotypes more effectively and consistently than previous models by combining yield and stability derived from WAASB. Similar findings have been reported by Zeeshan et al. [55], where they applied multiple models for identification of stable high yielder wheat genotypes across multiple environments. They advocated that although AMMI model is robust, but WAASBY outperforms it since it categorizes the genotypes into groups based on their stability and productivity performance. Hussain et al. [56] also found WAASB helpful in identifying high-yielding and stable chickpea genotypes for drought-susceptible regions. Similarly, comparative analyses in rice breeding indicated that the integration of yield with the WAASBY index achieved higher genetic gains than traditional approaches such as the GGE bi-plot, Relative Performance of Genotypic Values (RPGV), and Harmonic Mean of Genotypic Values (HMGV) models [57]. Furthermore, in the evaluation of crops like purple corn and chickpea, the WAASBY framework provided more robust selection criteria and superior multi-trait selection outcomes compared to utilizing AMMI or GGE bi-plots in isolation [58].

The Genotype Selection Index (GSI), which aggregates rankings from multiple models, also supported G8, G4, and G9 as superior genotypes. Spearman’s rank correlations confirmed WAASBY’s reliability, showing strong concordance with other indices. These results align with broader recommendations advocating multi-model stability analyses for robust genotype selection.

While the integration of multiple stability models offers a comprehensive approach to genotype selection, it is crucial to acknowledge the limitations inherent in each model. The effectiveness of these models can be influenced by the specific environmental conditions of the study, as seen in the varying productivity across different environments [25]. The WAASBY index offers a practical and powerful option for balancing yield and stability, functioning both as a complement to multi-model frameworks and as a standalone selection tool [59]. Genotypes G8, G4, and G9 consistently emerged as top candidates, highlighting their potential for further testing and varietal release.

Although the present study evaluated lentil genotypes across four diverse environments and detected significant genotype × environment interactions, the relatively small number of test environments is still a limitation. Four environments may not fully capture the range of climatic and stress conditions experienced by lentil across different production regions and years. Therefore, the stability and adaptability of the selected genotypes should be further validated through multi-year and multi-location trials, including environments with contrasting environmental conditions. The identification of stable genotypes also depends on the availability of sufficient genetic variability within the breeding material. Evidence from other crops supports the importance of exploiting genetic variation for developing stress-adapted cultivars. Murtaza et al. [60] reported significant genetic variability among bitter gourd parents and hybrids and identified promising hybrids for further evaluation, highlighting the value of genetic diversity in crop improvement. Similarly, Sattar et al. [61] identified diverse double-haploid maize lines with good performance under both normal and high-temperature conditions, demonstrating the importance of screening breeding material under target stress conditions to identify adaptable genotypes. These findings support the need to combine multi-environment stability analysis with targeted stress screening in lentil breeding programs to identify genotypes that maintain high yield and stable performance under increasingly variable climatic conditions.

## Conclusion

The present investigation highlighted substantial environmental influence on lentil yield performance, confirming that stability assessment across locations is essential for reliable genotype selection. The comparative use of different analytical models provided complementary insights into genotype behaviour under variable conditions. Regression-based analysis identified consistent performers, while multivariate models such as AMMI and BLUP effectively captured complex interaction patterns and differentiated genotypes with specific or broad adaptability. The WAASBY model further strengthened selection efficiency by integrating yield potential and stability into a single index, facilitating balanced genotype ranking. Together, these models revealed several genotypes with both superior productivity and consistent performance across environments, representing valuable material for varietal release and hybridization programs. Overall, this study demonstrates that applying multiple stability models enhances selection precision and provides a holistic understanding of genotype performance, thereby supporting the development of lentil cultivars resilient to environmental fluctuations and suitable for sustainable production systems.

## Supporting information

S1 Table Pooled Analysis of variance (ANOVA) of yield data of 14 genotypes across 04 environments (two years at two locations). (DOCX)
