# Supplementary material for: Multi-model assessment of yield stability in lentil across multi-environments
Source: PLoS One. 2026 Sep 1;21(9):e0356741. doi: 10.1371/journal.pone.0356741 (PMC13533361; doi:10.1371/journal.pone.0356741)
Supplement: Supplementary file 1 [file pone.0356741.s001.docx]

**Supp. Table 1.** **Pooled Analysis of variance (ANOVA) of yield data of 14 genotypes across 04 environments (two years at two locations)**

| Source | Df | Sum Sq | Mean Sq | F value |
| --- | --- | --- | --- | --- |
| ENV | 3 | 10567376.86 | 3522458.95 | 56.04 *** |
| REP(ENV) | 8 | 920871.04 | 115108.88 | 1.83 |
| GEN | 13 | 7051189.62 | 542399.20 | 8.63*** |
| GEN: ENV | 39 | 6023745.50 | 154455.01 | 2.46*** |
| Residuals | 104 | 6537495.61 | 62860.53 |  |
| CV (%) | 22.31 |  |  |  |
| OVmean | 1123.61 |  |  |  |

Df: degree of freedom, Sq: squares, ENV: Environment, GEN: Genotypes, REP: Replication,

CV: coefficient of variation, Significance Codes: ‘***’ 0.001 ‘**’ 0.01 ‘*’ 0.05
